# Parkin-deficient mice are not more sensitive to 6-hydroxydopamine or methamphetamine neurotoxicity

**DOI:** 10.1186/1471-2202-6-71

**Published:** 2005-12-24

**Authors:** Francisco A Perez, Wendy R Curtis, Richard D Palmiter

**Affiliations:** 1 Graduate Program in Neurobiology and Behavior, Medical Scientist Training Program, University of Washington, Seattle, WA 98195, USA; 2 Department of Biochemistry, Howard Hughes Medical Institute, University of Washington, Seattle, WA 98195, USA

## Abstract

**Background:**

Autosomal recessive juvenile parkinsonism (AR-JP) is caused by mutations in the *parkin *gene which encodes an E3 ubiquitin-protein ligase. Parkin is thought to be critical for protecting dopaminergic neurons from toxic insults by targeting misfolded or oxidatively damaged proteins for proteasomal degradation. Surprisingly, mice with targeted deletions of *parkin *do not recapitulate robust behavioral or pathological signs of parkinsonism. Since Parkin is thought to protect against neurotoxic insults, we hypothesized that the reason Parkin-deficient mice do not develop parkinsonism is because they are not exposed to appropriate environmental triggers. To test this possibility, we challenged Parkin-deficient mice with neurotoxic regimens of either methamphetamine (METH) or 6-hydroxydopamine (6-OHDA). Because Parkin function has been linked to many of the pathways involved in METH and 6-OHDA toxicity, we predicted that Parkin-deficient mice would be more sensitive to the neurotoxic effects of these agents.

**Results:**

We found no signs consistent with oxidative stress, ubiquitin dysfunction, or degeneration of striatal dopamine neuron terminals in aged Parkin-deficient mice. Moreover, results from behavioral, neurochemical, and immunoblot analyses indicate that Parkin-deficient mice are not more sensitive to dopaminergic neurotoxicity following treatment with METH or 6-OHDA.

**Conclusion:**

Our results suggest that the absence of a robust parkinsonian phenotype in Parkin-deficient mice is not due to the lack of exposure to environmental triggers with mechanisms of action similar to METH or 6-OHDA. Nevertheless, Parkin-deficient mice could be more sensitive to other neurotoxins, such as rotenone or MPTP, which have different mechanisms of action; therefore, identifying conditions that precipitate parkinsonism specifically in Parkin-deficient mice would increase the utility of this model and could provide insight into the mechanism of AR-JP. Alternatively, it remains possible that the absence of parkinsonism in Parkin-deficient mice could reflect fundamental differences between the function of human and mouse Parkin, or the existence of a redundant E3 ubiquitin-protein ligase in mouse that is not found in humans. Therefore, additional studies are necessary to understand why Parkin-deficient mice do not display robust signs of parkinsonism.

## Background

Parkinson's disease (PD) is a common neurological disorder that leads to severe motor disabilities due to degeneration of dopaminergic neurons in the substantia nigra pars compacta. Although it remains unclear how or why these specific neurons degenerate, the mechanisms likely depend on both environmental and genetic factors.

Epidemiological studies suggest that exposure to certain environmental factors, such as pesticides, well water, heavy metals, solvents, or carbon monoxide, significantly increase the risk of developing PD [1-4]. These environmental factors are thought to inhibit mitochondrial function, increase oxidative stress, damage proteins, and inhibit ubiquitin-proteasome system (UPS) function in dopamine neurons [5].

Genetic studies have determined that mutations in the human *parkin *gene can cause AR-JP, a PD-like disorder [6]. The Parkin protein is a widely expressed, E3 ubiquitin-protein ligase that is thought to target proteins, possibly those oxidatively damaged from environmental insults, for proteasomal degradation [6-8]. In the absence of Parkin function, oxidatively damaged proteins may not be cleared by the cell [9], and these proteins could in turn propagate damage to additional proteins [10]. Parkin may also be involved in the unfolded protein response (UPR) to endoplasmic reticulum (ER) stress [8,11] or in maintaining mitochondrial function [12,13]. Dysfunction in either of these pathways as a result of *parkin *mutations could also cause oxidative stress and cell death [14,15]. Understanding how mutations in *parkin *lead to dopamine neuron cell death could provide insight into the mechanism and treatment of other PD-like disorders.

To investigate how mutations in *parkin *lead to parkinsonism, several lines of mice with a targeted disruption of the mouse *parkin *gene, *Park2*, have been generated. Given the overwhelming evidence that mutations in *parkin *cause AR-JP in humans, it is surprising that mice with targeted deletions of *parkin *do not develop robust behavioral or pathological signs of parkinsonism [16-19]. These findings suggest that loss of Parkin function may not be sufficient for dopamine neuron degeneration in mice. Consistent with this idea, the age of onset, disease progression, and symptom severity can differ between AR-JP patients with similar *parkin *mutations, suggesting that environmental factors may contribute to the pathogenesis [20-22]. Since Parkin is thought to protect against neurotoxic insults [23], we hypothesized that the reason Parkin-deficient mice do not develop parkinsonism is because they are not exposed to appropriate environmental stress.

In this study, we extended our initial characterization study [16] by specifically investigating whether aged Parkin-deficient mice exhibit signs of ubiquitin dysregulation, increased oxidative damage to proteins, or dopamine terminal degeneration consistent with Parkin's proposed function in humans. We also evaluated whether Parkin-deficient mice are more sensitive to nigrostriatal injury following treatment with the neurotoxins 6-OHDA or METH.

6-OHDA is a hydroxylated dopamine analogue that is readily oxidized causing ROS-mediated damage [24], mitochondrial dysfunction [25], ER stress, activation of the UPR [26,27], and an increase in ubiquitin-mediated protein degradation [28]. Some of these effects are likely mediated by iron-dependent mechanisms [29,30]. Moreover, 6-OHDA toxicity is exacerbated by ubiquitin-proteasome system dysfunction [28], and Parkin-overexpression in cell culture can protect against 6-OHDA toxicity [31].

METH is a drug of abuse that releases vesicular dopamine into the cytoplasm and extracellular space [32]. The displaced dopamine can be readily oxidized [33] and may underlie the observed increases in hydroxyl radical levels [34], oxidatively damaged proteins [35], and endogenous formation of 6-OHDA [36] following METH treatment. METH neurotoxicity can inhibit mitochondrial function [37,38] and can be modified by alterations in mitochondrial function [39,40]. Moreover, METH toxicity involves autophagy [41], resembles ubiquitin-proteasome system dysfunction [42], and can activate ER stress and mitochondrial cell-death pathways [43]. There is also evidence that METH toxicity is partially mediated by glutamate [44,45].

Because Parkin function has been linked to many of the pathways involved in METH and 6-OHDA toxicity, we predicted that Parkin-deficient mice would be more sensitive to the neurotoxic effects of these agents.

## Results

### Aged Parkin-deficient mice do not exhibit signs consistent with ubiquitin dysfunction, oxidative stress, or dopamine neuron terminal degeneration

We predicted that the absence of Parkin in mice would lead to dysregulation of total ubiquitin levels; however, there was no difference between aged, 22-month-old wild-type control mice (WT) and Parkin-deficient mice (KO) using immunoblot analysis of striatal or cortical tissue (Table 1). We also hypothesized that Parkin-deficient mice would exhibit an accumulation of oxidized proteins. Oxidation of proteins by reactive oxygen species and other reactive species, such as 4-hydroxy-2-nonenal from lipid peroxidation, produces protein carbonyl derivates [46]. Protein carbonyl levels have been extensively used as a sensitive marker for oxidative stress in various model systems [46], and patients with PD exhibit a general increase in protein carbonyls [47]. Using immunoblot analysis, we detected no differences in the levels of striatal or cortical protein carbonyls between aged WT and KO mice (Table 1). To evaluate dopamine neuron terminal integrity in Parkin-deficient mice, we performed immunoblot analysis of striatal dopamine transporter (DAT) levels. No difference between WT and KO mice was detected (Table 1).

### Parkin-deficient mice are not more sensitive to 6-OHDA toxicity

To determine whether Parkin-deficient mice are more sensitive to the neurotoxin 6-OHDA, we treated 3-month-old WT and KO mice with a unilateral, intrastriatal injection of saline (SAL) or 6-OHDA. Mice were treated with either a 4 μg or 8 μg dose of 6-OHDA because our objective was to find a dose that would cause mild neurotoxicity in WT mice but severe neurotoxicity in KO mice.

**Figure 1:**
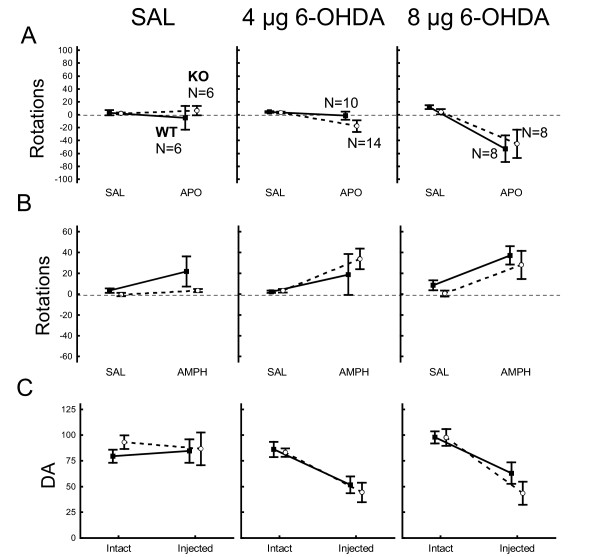
**Parkin deficient-mice are not more sensitive to 6-OHDA toxicity. A. **WT mice (filled square symbols) and KO mice (open circle symbols) treated with 4 μg 6-OHDA or 8 μg 6-OHDA displayed similar contralateral, rotational behavior (expressed as net negative rotations) following APO-treatment. **B. **Ipsilateral rotational behavior (expressed as net positive rotations) following AMPH treatment was also indistinguishable between WT and KO mice. **C. **There was no difference between WT and KO mice in striatal dopamine (DA) levels (expressed as ng/mg protein) on the intact, uninjected side versus the side treated with SAL, 4 μg 6-OHDA, or 8 μg 6-OHDA. The numbers of mice used for each genotype are indicated in A; these same mice were also analyzed in B and C. Data are expressed as mean ± SEM.

Beginning 14 days after surgery, analyses of apomorphine (APO)- and amphetamine (AMPH)- induced rotational behaviors were conducted to evaluate the consequences of 6-OHDA neurotoxicity [48]. Mice were first challenged with the postsynaptic dopamine receptor agonist APO. Rotations away from the lesioned side (contralateral) reflect postsynaptic dopamine receptor supersensitivity following striatal dopamine depletion [49,50]. Repeated measures ANOVAs were used to compare the number of net rotations following a subcutaneous injection of SAL versus APO. There was no significant effect of APO on rotational behavior in mice that received an intrastriatal injection of SAL; however, mice treated with 4 or 8 μg 6-OHDA demonstrated significant contralateral rotations (4 μg 6-OHDA, *F*_1,22 _= 4.6, *p *= 0.04; 8 μg 6-OHDA, *F*_1,14 _= 12.7, *p *= 0.003). Moreover, there was a dose-dependent effect of 6-OHDA on rotational behavior; mice treated with 8 μg 6-OHDA exhibited significantly greater contralateral rotations compared to mice treated with 4 μg 6-OHDA (APO Treatment × 6-OHDA Treatment interaction, *F*_1,36 _= 8.7, *p *= 0.006). However, there was no difference in APO-induced rotational behavior between WT and KO mice treated with SAL or either dose of 6-OHDA (Fig. 1A; Genotype × APO Treatment interaction: 4 μg 6-OHDA, *F*_1,22 _= 1.5, *p *= 0.24; 8 μg 6-OHDA, *F*_1,14 _= 0.2, *p *= 0.65).

Fifteen days after surgery, mice were challenged with AMPH to evaluate rotational behavior. Because AMPH causes presynaptic dopamine release, rotations towards the lesioned side (ipsilateral) reflect loss of striatal dopamine terminals [49,50]. AMPH had no effect on rotational behavior in mice that received an intrastriatal injection of SAL (*F*_1,10 _= 2.9, *p *= 0.12). Mice that received 4 or 8 μg 6-OHDA demonstrated significant ipsilateral rotations following AMPH treatment (4 μg 6-OHDA, *F*_1,22 _= 5.8, *p *= 0.02; 8 μg 6-OHDA, *F*_1,14 _= 16.9, *p *= 0.001); however, a dose-dependent effect of 6-OHDA on AMPH-induced rotations was not observed. There was no difference between WT and KO mice in AMPH-induced rotational behavior at either dose (Fig. 1B; Genotype × AMPH Treatment interaction: 4 μg 6-OHDA, *F*_1,22 _= 0.5, *p *= 0.49; 8 μg 6-OHDA, *F*_1,14 _= 0.0, *p *= 0.93).

Eighteen days after surgery, striatal samples from the injected and uninjected sides were collected for neurochemical analyses to determine the concentrations of the neurotransmitters norepinephrine (NE), dopamine, and serotonin (5-HT); the dopamine-related metabolites 3-methoxytyramine (3-MT), 3,4-dihydroxyphenylacetic acid (DOPAC), and homovanillic acid (HVA); and the 5-HT-related metabolite 5-hydroxyindoleacetic acid (5-HIAA). For each animal, data from the treated (injected) striatum was compared to data from the untreated striatum using repeated measures ANOVAs. Intrastriatal injection of SAL had no effect on dopamine levels (*F*_1,10 _= 0.0, *p *= 0.95) or any other neurochemicals measured (3-MT, DOPAC, HVA, NE, 5-HT, 5-HIAA). Treatment with 4 μg or 8 μg 6-OHDA led to a ~40% reduction in dopamine levels (4 μg 6-OHDA, *F*_1,22 _= 24.0, *p *< 0.0001; 8 μg 6-OHDA, *F*_1,14 _= 14.8, *p *= 0.002); however, there was no difference between WT and KO mice at either dose (Fig. 1C; Genotype × 6-OHDA Treatment interaction: 4 μg 6-OHDA, *F*_1,22 _= 0.1, *p *= 0.78; 8 μg 6-OHDA, *F*_1,14 _= 0.7, *p *= 0.41). Although treatment with 8 μg 6-OHDA led to more APO-induced rotational behavior compared to 4 μg 6-OHDA, it did not produce a significantly greater dopamine depletion (*F*_1,36 _= 0.4, *p *= 0.54). Significant reductions in DOPAC, HVA, 3-MT, and dopamine/DOPAC were observed in striata treated with 6-OHDA; Genotype had no effect on these decreases (data not shown). 5-HT or 5-HIAA levels were not reduced on the 6-OHDA-lesioned side, but there was a trend towards decreased NE on the lesioned side with no effect of Genotype (data not shown).

### Parkin-deficient mice are not more sensitive to METH toxicity

To determine whether Parkin-deficient mice are more sensitive to the neurotoxin METH, 3-month-old WT and KO mice were treated with repeated, acute administration of SAL or METH. Mice received either a low (2.5 mg/kg body weight) or moderate dose (5.0 mg/kg body weight) of METH because our objective was to find a dose that would cause mild neurotoxicity in WT mice but severe neurotoxicity in KO mice.

**Figure 2:**
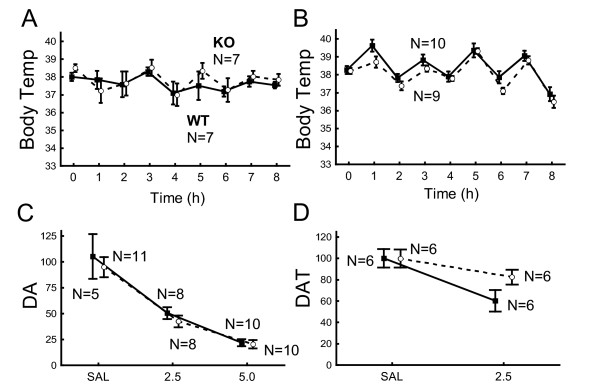
**Parkin-deficient mice are not more sensitive to METH toxicity. **The hyperthermic response to METH treatment at **A. **2.5 mg/kg and **B. **5.0 mg/kg was indistinguishable between WT (filled square symbols) and KO (open circle symbols) mice. Body temperature (°C) was measured before METH treatment (time = 0 hr) and every hour thereafter. **C. **There was no difference between WT and KO mice in the depletion of striatal dopamine (DA) levels (expressed as ng/mg protein) following METH treatment. **D. **There was also no difference in the reduction of striatal DAT levels following METH treatment (data are expressed in arbitrary units after normalization to WT levels). The numbers of mice used for each genotype are indicated in the figure. Data are expressed as mean ± SEM.

We have previously demonstrated that KO mice have a normal hyperlocomotor response to repeated administration of AMPH (4.0 mg/kg body weight), a psychostimulant similar to METH [16]. Because METH neurotoxicity can be modified by decreasing METH-induced hyperthermia [51], differences between WT and KO mice in body temperature regulation would confound interpretation of results. Body temperature regulation over 24 hr and in response to a cold challenge is indistinguishable between aged WT and KO mice; moreover, there are no differences in body temperature between 3-month-old WT and KO mice [16]. In the present study, we monitored body temperature before and during METH treatment in WT and KO mice. Treatment with METH at 5.0 mg/kg led to a significantly greater METH-induced hyperthermia compared to treatment with 2.5 mg/kg (Dose effect, *F*_1,29 _= 7.1, *p *= 0.01); however, there were no significant body temperature differences between WT and KO mice following METH treatment (Fig. 2A, 2B; Genotype effect, *F*_1,29 _= 0.3, *p *= 0.56; Genotype × Dose interaction, *F*_1,29 _= 2.4, *p *= 0.13). The mean body weight among all 3-month-old WT and KO mice used for this study was not different (WT = 32.0 ± 0.4 g, N = 47; KO = 31.7 ± 0.5 g, N = 57; Student t-test, *p *= 0.69).

Seven days following treatment, METH neurotoxicity was evaluated by neurochemical and immunoblot analyses. There was a significant reduction in striatal dopamine levels following METH treatment (Treatment effect, *F*_2,46 _= 45.4, *p *< 0.0001). Treatment with METH at 5.0 mg/kg produced a significantly greater dopamine depletion compared to 2.5 mg/kg (80% versus 50% dopamine reduction; *F*_1,32 _= 30.0, *p *< 0.0001). However, we detected no difference between genotypes in the magnitude of dopamine depletion following METH treatment (Fig. 2C; Genotype effect, *F*_1,46 _= 0.9, *p *= 0.34; Genotype × Treatment interaction, *F*_2,46 _= 0.2, *p *= 0.86). We also observed significant reductions in the dopamine metabolites DOPAC, HVA, and 3-MT following METH treatment, but there were no differences between genotypes in these reductions (data not shown). Reductions in striatal NE, 5-HT, or 5-HIAA were not detected (data not shown). Immunoblot analysis of striatal DAT levels, a marker for dopamine neuron terminal integrity, demonstrated a 30% reduction following treatment with METH (2.5 mg/kg; Treatment effect, *F*_1,20 _= 10.3, *p *= 0.004); however, there was no difference between WT and KO mice (Fig. 2D; Genotype × Treatment interaction, *F*_1,20 _= 1.6, *p *= 0.22).

## Discussion

We expected Parkin-deficient mice to demonstrate increased signs of oxidized proteins as a result of increased pro-oxidant activity, for example due to mitochondrial dysfunction; decreased antioxidant activity, for example due to dysregulation of glutathione levels; or decreased protein clearance due to dysfunction of the ubiquitin proteasome system. Previous studies have identified increased protein carbonyls in the brains of Parkin-deficient mice [13], a slight increase in GSH levels specifically in the striatum, and an increase in DOPAC/3-MT levels consistent with a shift towards dopamine metabolism by the H_2_O_2_-producing monoamine oxidase pathway [18]. A slight reduction in striatal DAT levels was also previously observed, which may reflect initial damage to dopamine neuron terminals [18]. In this study, Parkin-deficient mice did not exhibit increased levels of protein carbonyls, and consistent with our previous report using aged mice [16], we found no difference in DOPAC/3-MT levels between young WT and KO mice (data not shown). Moreover, levels of total ubiquitin and striatal DAT in aged Parkin-deficient mice were indistinguishable from WT mice. Our results suggest that loss of Parkin in mice does not lead to robust oxidative stress, ubiquitin dysfunction, or dopamine neuron terminal degeneration. Nevertheless, we cannot rule out the possibility that differences in oxidation between WT and KO mice are limited to a few specific proteins [52].

The current study extends the number of inconsistent findings reported in several Parkin-deficient mouse models [13,16-19]. Reasons for discrepancies among these Parkin-deficient mouse models have been previously discussed and include differences in experimental technique, differences in genetic background, differences in the gene targeting strategy, or confounds of gene targeting [16]. Of biological significance, the type of targeted mutation in these mouse models could differentially affect expression of potential Parkin isoforms with unique functions [53-55]. Parkin-deficient mice used in this study lack exon 2, which encodes most of the ubiquitin-like domain believed to be required for interacting with the proteasome [56] and ubiquitinated substrates [57].

Interpretation of studies using Parkin-deficient mice can be complicated because a mixed B6;129 genetic background was used in this study and most previous studies; therefore, many genes linked to *parkin *will be 129 derived in KO mice and B6 derived in WT mice [16]. Even after 12 backcrosses to the B6 genetic background, ~16 cM surrounding the mutant *parkin *allele could remain 129 derived; however, in control mice the corresponding region will likely remain B6 derived [58,59]. It is possible that the increased oxidative stress and protein carbonyls previously reported in Parkin-deficient mice are not due to the *parkin *mutation but reflect strain differences between WT and KO mice in these closely linked genes. For example, *mitochondrial superoxide dismutase 2 *(*Sod2*), which is tightly linked to *parkin *(~2 cM [60]), is involved in mitochondrial function and protects against oxidative stress. *Sod2 *polymorphisms between inbred mouse strains have been described and can lead to a reduction in specific activity of the protein and an increase in protein carbonyls [61,62]. It is very likely that the *Sod2 *gene will differ between Parkin-deficient and control mice unless appropriate breeding strategies are employed.

Despite the inconsistencies among studies involving Parkin-deficient mice, we sought to determine whether there were any functional consequences of Parkin-deficiency following neurotoxic treatment with 6-OHDA or METH. Parkin-deficient mice were not more sensitive to 6-OHDA neurotoxicity. Specifically, the dose-dependent, behavioral response to 6-OHDA treatment was indistinguishable between WT and KO mice. Moreover, because APO-induced rotational behavior reflects dopamine-receptor supersensitivity [49,50], our results suggest that the compensatory mechanisms underlying this phenomenon are intact in Parkin-deficient mice. Despite the dose-dependent increase in APO-induced rotational behavior, we were unable to detect a dose-dependent effect of 6-OHDA on dopamine depletion, thereby supporting a role for variables other than the extent of dopamine depletion on APO-induced rotation [49,50]. With regard to the role of Parkin in 6-OHDA toxicity, it has been observed that overexpression of Parkin protects against 6-OHDA toxicity in some cell culture models [31] but not in others [63]. Our results suggest that Parkin-deficiency in mice does not dramatically affect the pathways involved in 6-OHDA neurotoxicity.

We previously reported that locomotion following treatment with AMPH, which is chemically related to METH, is indistinguishable between WT and KO mice [16]; in this study we demonstrate that there is no difference in METH-induced hyperthermia. Previous studies have suggested that mouse Parkin is involved in regulating dopaminergic and glutamatergic neurotransmission [18]. Our pharmacological, behavioral, neurochemical, and toxicity studies are not consistent with these findings [16]. It was also shown that Parkin can protect dopamine neurons in a cell-culture model of glutamate excitotoxicity [64]. Our results indicate that this finding cannot be extended to METH-related glutamate toxicity of dopaminergic neurons in a Parkin-deficient mouse model [44,45]. It has also been proposed that Parkin is involved in METH toxicity because Parkin-immunoreactive aggregates have been observed [42] and levels of Parkin and Pael-R, a putative target for Parkin-mediated ubiquitination, are decreased following METH treatment [65]. We found that Parkin was not essential for METH or 6-OHDA neurotoxicity.

Although our results suggest that Parkin-deficiency in young mice does not greatly modify susceptibility of dopaminergic neurons to acute METH or 6-OHDA neurotoxicity, interpretation is complicated by our use of a mixed genetic background. We cannot rule out the possibility that strain differences in genes linked to the targeted *parkin *allele may confer protection against toxicity specifically in KO mice thereby masking an effect due to Parkin-deficiency. For example, genes linked to *parkin*, such as *tumor necrosis factor *(~13 cM), are known to modify METH toxicity [66]. To avoid this complication, future work should utilize coisogenic mice as controls, especially when small differences between genotypes are detected. Despite this potential confound, Parkin does not appear to play a critical role in protection against METH or 6-OHDA neurotoxicity in mice.

Our results do not rule out the possibility that Parkin-deficient mice could be more sensitive to other neurotoxins which have different mechanisms of action than METH or 6-OHDA. For example, Parkin-deficient mice could be more sensitive to the neurotoxic effects of rotenone, maneb, paraquat, MPTP, or genetic overexpression of putative Parkin substrates, such as aminoacyl-tRNA synthetase cofactor p38 [67]. Moreover, interpretation of our results is limited by the specific neurotoxic regimens used in our experiments and the nature of negative findings; we cannot exclude the possibility that alternative METH or 6-OHDA neurotoxic regimens could reveal a latent susceptibility of Parkin-deficient mice to these agents. For example, our study utilized an acute model of toxicity in young mice; Parkin may protect dopaminergic neurons from specific, chronic insults, particularly in aged mice. Identifying conditions that precipitate parkinsonism specifically in Parkin-deficient mice would increase the utility of this model and could provide insight into the mechanism of AR-JP.

Alternatively, despite the limitations of our study, the findings that Parkin-deficient mice are not more sensitive to METH or 6-OHDA could suggest that the absence of a robust parkinsonian phenotype in Parkin-deficient mice is not due to the lack of exposure to appropriate environmental triggers. Parkin function in mice could be fundamentally different than in humans, or a redundant E3 ubiquitin-protein ligase, not found in humans, could exist in mice. Therefore, additional studies are necessary to understand why Parkin-deficient mice do not display robust signs of parkinsonism.

## Conclusion

Aged Parkin-deficient mice do not display signs consistent with oxidative stress, ubiquitin dysfunction, or dopamine neuron terminal degeneration. Moreover, 3-month-old Parkin-deficient mice on a mixed B6;129 genetic background are not more sensitive to the acute neurotoxic effects of METH or 6-OHDA.

## Methods

### Mice

Parkin-deficient mice, with a targeted deletion of *parkin *exon 2 (*Park2*^*tm1Rpa*^*/Park2*^*tm1Rpa*^; KO), and wild-type control mice (*Park2*^+^*/Park2*^+^; WT) were obtained by crossing *Park2*^*tm1Rpa*^*/Park2*^+ ^mice on a mixed B6;129S4 genetic background [16]. Twenty-two-month old, male mice were used for analysis of aged tissue; 3-month old, male mice were used for METH and 6-OHDA toxicity experiments. Mice were housed in a specific-pathogen-free facility, maintained on a 12-hr light cycle, housed in groups, and provided with free access to water and food (5053, LabDiet; Richmond, IN). All procedures adhered to the *Guide for the Care and Use of Laboratory Animals *[68] and were approved by the University of Washington Institutional Animal Care and Use Committee.

### 6-OHDA treatment

Mice received a unilateral, intrastriatal injection of SAL or a freshly prepared 6-OHDA solution (6-hydroxydopamine hydrobromide; Sigma-Aldrich; St. Louis, MO) using a stereotaxic surgical procedure [69,70]. 6-OHDA-treated mice received either a 4 μg or 8 μg dose, and all treatment preparations contained 0.02% ascorbic acid. Injections were targeted to the central caudate putamen using the following coordinates: 0.8 mm anterior to bregma, 2.0 mm lateral to bregma, and 3.6 mm ventral to the skull surface. Treatments were administered in a volume of 2.0 uL at a rate of 0.5 uL/min; after the injection, the needle was left in place for 5 min. Surgical treatments were performed without knowledge of genotypes. Fourteen and 15 days after surgery, rotational-behavior was evaluated in response to APO (apomorphine hydrochloride hemihydrate; 0.5 mg/kg body weight subcutaneously; Sigma-Aldrich) or AMPH (D-amphetamine sulfate salt; 2 mg/kg body weight intraperitoneally; Sigma-Aldrich), respectively. Individual mice were acclimated in a 16-cm diameter cylinder for 10 min. Mice were then administered SAL (either subcutaneously or intraperitoneally prior to APO or AMPH treatment, respectively), and the number of complete clockwise or counter-clockwise rotations were observed for 30 min. Mice were then administered either APO or AMPH and rotational behavior was scored for an additional 30 min. Rotational behavior was evaluated without knowledge of treatment or genotype. Eighteen days after surgery, striatal tissue was collected, as described [16], from both the injected and intact sides to evaluate integrity of dopamine-neuron terminals using neurochemical analyses.

### METH treatment

Mice were administered repeated, intraperitoneal injections of SAL or METH ((+)-methamphetamine hydrochloride; Sigma-Aldrich) every 2 hr for a total of 4 injections. METH-treated mice received either a low (2.5 mg/kg body weight for each injection) or moderate (5.0 mg/kg) neurotoxic regimen. Body temperature was monitored every hour during METH treatment using a subcutaneously implanted, programmable, temperature transponder (Bio Medic Data Systems; Seaford, DE). Seven days after treatment, striatal tissue was harvested to assess integrity of dopamine-neuron terminals using neurochemical and immunoblot analyses. Treatments were administered and tissue was collected without knowledge of genotypes.

### Neurochemical analyses

HPLC with electrochemical detection was used, as described [16], to determine the striatal concentrations of NE, dopamine, 5-HT, 3-MT, DOPAC, HVA, and 5-HIAA. Analyses were conducted without knowledge of treatment or genotype.

### Immunoblot analyses

Protein lysates from dissected mouse brain tissue were prepared by sonication in a solution containing 62.5 mM Tris-HCl (pH 6.8), 2% sodium dodecyl sulfate, 2% β-mercaptoethanol, Complete Protease Inhibitor Cocktail (Roche Diagnostics; Indianapolis, IN), and 1 mM phenylmethylsulfonyl fluoride. Samples were centrifuged (15,000 × *g*, 10 min) and the supernatant was used for immunoblot analysis. Protein (2 μg) was transferred to a PVDF membrane (Hybond-P; Amersham Biosciences; Piscataway, NJ) in duplicate using a slot-blot system (Bio-Rad Bio-Dot SF; Hercules, CA). A slot-blot approach was utilized because it enabled simultaneous analysis of a large number of samples and unbiased normalization using a fluorescent stain for total protein. For each sample, one replicate was subjected to immunoblot analysis, and the other replicate was analyzed for total protein using SYPRO Ruby Protein Blot stain (Molecular Probes; Eugene, OR). Membranes were blocked with 5% milk in TTBS (50 mM Tris-HCl, pH 7.5, 0.9% NaCl, 0.5% Tween20) for 1 hr, incubated with primary antibody diluted in blocking solution overnight (1:2000 rabbit anti-ubiquitin, Dako, Carpinteria, CA; or 1:10000 rat anti-DAT, Chemicon, Temecula, CA), washed 3 × 10 min in TTBS, incubated with HRP-conjugated secondary antibodies diluted in blocking solution for 1 hr (1:5000 anti-rabbit, Amersham Biosciences; 1:20000 anti-rat, Jackson ImmunoResearch Laboratories, Inc., West Grove, PA), washed 3 × 10 min in TTBS, and developed using ECL Plus (Amersham Biosciences, Piscataway, NJ). Blots were scanned using the Storm 840 system and quantitated using ImageJ (developed at the U.S. National Institutes of Health and available on the Internet at ). Oxidatively modified proteins were detected as described by Ballesteros et al. [71] using the OxyBlot Kit (Chemicon).

### Statistics

Data were analyzed using multi-way ANOVAs. Residuals and homogeneity of variance were evaluated to ensure the data met assumptions required for ANOVA. Data obtained from the same mouse, for instance before and after a treatment, were evaluated as a repeated measure. Data are presented as mean ± SEM.

## Authors' contributions

FAP developed the Parkin-deficient mice; conceived, designed, and carried out the study; analyzed and interpreted the data; and drafted the manuscript. WC performed animal surgeries and scored rotational behavior. RP participated in the design and coordination of the study. All authors read and approved the final manuscript.

## Figures and Tables

**Table 1:** Immunoblot analyses of aged Parkin-deficient mouse brains. Data are expressed as mean ± SEM in arbitrary units after normalization to WT levels.

**Immunoblot**	**Region**	**WT (N = 6)**	**KO (N = 6)**
Protein carbonyl	Striatum	100 ± 5	100 ± 13
	Cortex	100 ± 8	89 ± 7
Ubiquitin	Striatum	100 ± 9	95 ± 7
	Cortex	100 ± 8	98 ± 7
DAT	Striatum	100 ± 4	99 ± 8
